# Guideline adaptation and implementation planning: a prospective observational study

**DOI:** 10.1186/1748-5908-8-49

**Published:** 2013-05-08

**Authors:** Margaret B Harrison, Ian D Graham, Joan van den Hoek, Elizabeth J Dogherty, Meg E Carley, Valerie Angus

**Affiliations:** 1 School of Nursing, Queen’s University, 78 Barrie Street, Kingston, Ontario, K7L 3N6, Canada; 2 School of Nursing, University of Ottawa, 451 Smyth Road, Ottawa, Ontario, K1N 6N5, Canada

**Keywords:** Knowledge to action, Practice guidelines, Evidence-informed practice, Knowledge activation, Guideline adaptation, Implementation planning

## Abstract

**Background:**

Adaptation of high-quality practice guidelines for local use has been advanced as an efficient means to improve acceptability and applicability of evidence-informed care. In a pan-Canadian study, we examined how cancer care groups adapted pre-existing guidelines to their unique context and began implementation planning.

**Methods:**

Using a mixed-methods, case-study design, five cases were purposefully sampled from self-identified groups and followed as they used a structured method and resources for guideline adaptation. Cases received the ADAPTE Collaboration toolkit, facilitation, methodological and logistical support, resources and assistance as required. Documentary and primary data collection methods captured individual case experience, including monthly summaries of meeting and field notes, email/telephone correspondence, and project records. Site visits, process audits, interviews, and a final evaluation forum with all cases contributed to a comprehensive account of participant experience.

**Results:**

Study cases took 12 to >24 months to complete guideline adaptation. Although participants appreciated the structure, most found the ADAPTE method complex and lacking practical aspects. They needed assistance establishing individual guideline mandate and infrastructure, articulating health questions, executing search strategies, appraising evidence, and achieving consensus. Facilitation was described as a multi-faceted process, a team effort, and an essential ingredient for guideline adaptation. While front-line care providers implicitly identified implementation issues during adaptation, they identified a need to add an explicit implementation planning component.

**Conclusions:**

Guideline adaptation is a positive initial step toward evidence-informed care, but adaptation (vs. ‘de novo’ development) did not meet expectations for reducing time or resource commitments. Undertaking adaptation is as much about the process (engagement and capacity building) as it is about the product (adapted guideline). To adequately address local concerns, cases found it necessary to also search and appraise primary studies, resulting in hybrid (adaptation plus de novo) guideline development strategies that required advanced methodological skills.

Adaptation was found to be an action element in the knowledge translation continuum that required integration of an implementation perspective. Accordingly, the adaptation methodology and resources were reformulated and substantially augmented to provide practical assistance to groups not supported by a dedicated guideline panel and to provide more implementation planning support. The resulting framework is called CAN-IMPLEMENT.

## Background

### Bridging the ‘Know-Do’ gap

‘Health work teaches us with great rigour that action without knowledge is wasted effort, just as knowledge without action is a wasted resource’ [1].

‘Canadian researchers are doing an exceptional job making discoveries and generating new knowledge that has the potential to improve the health of Canadians and strengthen Canada’s healthcare system and economy but unless this knowledge is actually put into action, these benefits will not be realized’ [2].

While the importance of ‘turning knowledge into action’ and using available evidence to inform practice is widely recognized, it presents a challenge to most healthcare jurisdictions. To explore how this occurs at the point of care, a pan-Canadian initiative followed five groups (‘cases’) as they undertook to adapt guidelines and begin implementation of research evidence in cancer care.

Practice guidelines form one important piece of the larger evidence-based practice puzzle. As a source of readily available evidence, rigorously synthesized and interpreted by expert clinicians and methodologists and transformed into practice recommendations, quality guidelines have the potential to improve both the process of care and patient outcomes. Over the past decade, a growing number of guideline entities have generated scores of guidelines. For example, the Canadian Partnership Against Cancer recently catalogued over 2,200 cancer care guidelines [3]. However, guideline duplication is common and guideline quality is variable [4]. Moreover, despite large-scale initiatives across disease and health conditions to develop these knowledge tools, their uptake in practice is not apparent. Producing good quality, readily available guidelines is no guarantee that the recommendations will be implemented in healthcare practice or policy. As Straus and colleagues summarize: ‘Failure to use research evidence to inform decision-making is apparent across all key decision-maker groups, including healthcare providers, patients, informal carers, managers and policy makers, in developed and under developed countries, in primary and specialty care, and in care provided by all disciplines’ [5]. It is clear that emphasis must shift from guideline development to guideline use. Turning to available evidence, housed in appropriate guidelines, is a potential solution (or part of the solution) and the process of guideline adaptation becomes the initial step in creating change.

In this paper, we present the experience of Canadian cancer care groups undertaking guideline adaptation and implementation planning. We describe the evolution of the ADAPTE methodology as it was used naturalistically in the field, the development of expanded resources, and the emergence of an implementation-based framework for knowledge translation with guidelines.

### The study opportunity

The Canadian Partnership Against Cancer (the ‘Partnership’) was established in 2006 as a federally-funded, independent corporation to develop and implement the first national cancer control strategy. Cancer control knowledge and expertise are widely dispersed throughout Canada’s healthcare system. To make this knowledge readily accessible to stakeholders, collaborative networks of experts were established to address multiple priorities, including knowledge translation and the role and use of guidelines. Given the duplication of guidelines by credible bodies internationally, jurisdictions within Canada were interested in the process of adapting guidelines to their own context.

Guideline adaptation is defined as the ‘systematic approach to considering the use and/or modification of (a) guideline(s) produced in one cultural and organizational setting for application in a different context’ [6]. Customizing evidence-informed guideline recommendations for local application demands both methodological expertise and an intimate knowledge of the intended clinical practice environment. Dedicated guideline development bodies may have greater capacity to synthesize evidence but often have limited access to detailed contextual information. The Partnership identified a need to explore how guideline adaptation occurs in real-world settings.

This initiative coincided with the 2007 first release of ADAPTE, a systematic, stepwise approach to trans-contextual guideline adaptation (Table 1) [6]. ADAPTE was developed by an international, interdisciplinary collaboration of guideline developers, researchers and clinicians. Evolving from research conducted in Canada and Europe [5-8], guideline adaptation was proposed as an efficient alternative to de novo guideline development, a method which requires skilled and labor-intensive meta-analysis of original research. Although promising as a technique for making use of already developed guidelines, the ADAPTE method had not been field-tested, in particular for use by groups lacking the resources or skills of commissioned guideline panels.

**Table 1:** The ADAPTE method (version 1.0)

**PHASE 1 SET-UP**	**MODULE**	**STEPS**
	**Prepare for ADAPTE process**	1. Establish an organizing committee
		2. Select a topic
		3. Check whether adaptation is feasible
		4. Identify skills and resources needed
		5. Complete set-up tasks
		6. Write protocol
**PHASE 2 ADAPTATION**	**MODULE**	**STEPS**
	**Scope and Purpose**	7. Determine the health questions
	**MODULE**	**STEPS**
	**Search and Screen**	8. Search for guidelines and other relevant documentation
		9. Screen retrieved guidelines
		10. Reduce total number of guidelines if there are more than can be dealt with by the panel
	**MODULE**	**STEPS**
	**Assessment**	11. Assess guideline quality
		12. Assess guideline currency
		13. Assess guideline content
		14. Assess guideline consistency (search and selection of studies, links between evidence and recommendations)
		15. Assess acceptability/applicability of the recommendations
	**MODULE**	**STEPS**
	**Decision and Selection**	16. Review assessments to aid in decision-making
		17. Select between guidelines and recommendations to create an adapted guideline
	**MODULE**	**STEPS**
	**Customization**	18. Prepare a document that respects the needs of the end users and provides a detailed transparent explanation of the process
**PHASE 3 FINALIZATION**	**MODULE**	**STEPS**
	**External Review and Acknowledgement**	19. External review by target users
		20. Consult with relevant endorsement bodies
		21. Consult with developers of source guidelines
		22. Acknowledge source documents
	**MODULE**	**STEPS**
	**Aftercare Planning**	23. Plan for aftercare of the adapted guideline
	**MODULE**	**STEPS**
	**Final Production**	24: Produce high quality final guideline

The Canadian Guideline Adaptation Study was designed to examine how groups used a ‘planned action’ approach to integrate knowledge created outside their context with local practice and system requirements. Canadian cancer care groups volunteered to embark on the adaptation of existing quality guidelines while allowing the study team at Queen’s University School of Nursing to follow their journey. The opportunity for naturalistic observation was endorsed by the Canadian Partnership Against Cancer with the proviso that participants would be supported as we monitored their guideline adaptation activity and experience. Using a formative rather than prescriptive approach, our aim was to track the practicalities encountered using the ADAPTE methodology (process evaluation), to provide needed support for them to successfully adapt a guideline (formative evaluation), and to develop experientially-informed, additional tools and resources as needed. These tools were to be housed with the Partnership for on-going use.

### Conceptualization in activating healthcare knowledge

Knowledge creation and application is an iterative, dynamic and complex process. The planned-action framework, Knowledge to Action (KTA) cycle [5,9], guided this initiative. The KTA cycle encompasses two major elements: knowledge creation and planned action [5,8]. Knowledge creation involves three stages to tailor and refine information for use: knowledge enquiry (*e*.*g*., primary studies), synthesis (*e*.*g*., a body of work, meta-analysis), and creation of knowledge tools or products (*e*.*g*., guidelines). Recognition of a gap in care serves as an important stimulus for action. Distilled knowledge is applied or set in motion when a care issue is identified, and this handover prompts a cycle of activity, sequentially or concurrently, requiring users to:

1. Identify and clarify the practice problem or issue(s) to be addressed;

2. Identify, review and select the knowledge (*i*.*e*., knowledge synthesis or knowledge product – *e*.*g*., guideline) that provides a solution to the identified problem;

3. Adapt or customize the knowledge to the local context (*i*.*e*., practice and system);

4. Assess local determinants of knowledge use (*i*.*e*., barriers and facilitating factors);

5. Select, tailor, and implement interventions to promote knowledge use (*i*.*e*., implement the change);

6. Monitor the uptake, evaluate the impact of using the knowledge, and sustain knowledge use.

**Figure 1:**
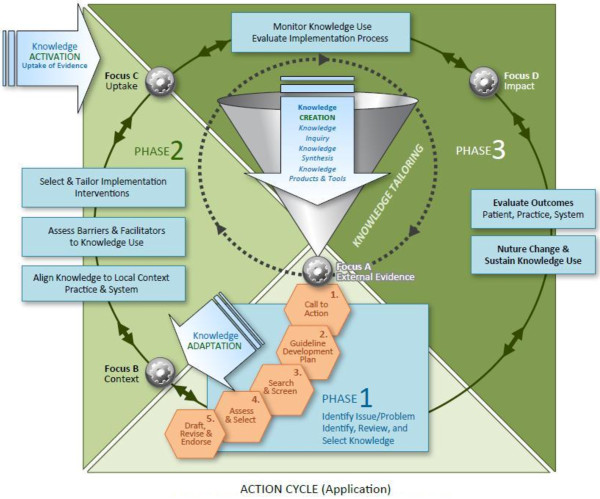
Knowledge to Action (KTA) Framework (Graham, Logan, Harrison, 2006) with integration of guideline adaptation; CAN-IMPLEMENT.

Knowledge creation and application are interconnected with fluid boundaries. Guideline adaptation may occur within the ‘knowledge creation funnel’ (Figure 1) when undertaken by guideline developers adapting an existing guideline(s) to create a modified/new guideline (*i*.*e*., a knowledge product or tool) for dissemination, *e*.*g*., a Canadian association adapting guidelines developed in the USA and UK for use in their jurisdiction. Guideline adaptation also occurs as a planned-action process when implementing evidence, *e*.*g*., groups work on the quality appraisal of existing guideline(s) and specific practice recommendations to use this evidence locally. Practice patterns, provider expertise, available resources, and patient population(s) are examined to actively engage in knowledge application and the initial steps in the action cycle.

The ADAPTE Collaboration recently published feedback from an on-line survey they conducted during the same period [10]. Survey participants could download the ADAPTE Manual and Toolkit but received no additional assistance; they did not necessarily engage in adaptation, and their activities were not monitored. Questions were directed primarily at ‘perceptions’ of the methodology and supporting resource package. The Canadian Guideline Adaptation Study provided a unique opportunity to closely observe and support adaptation and implementation planning activity in real time.

An additional, embedded study focused on facilitation aspects. The theoretical framework and findings for this element have been published in-depth elsewhere [11,12].

## Methods

The study design emerged during a six-month pilot case study that undertook a simple field evaluation of ADAPTE. Data management and evaluation protocols were developed and tested, the trajectory of steps adjusted, and significant needs for additional support addressed. We then began a formative assessment with additional cases. In this manner, the Canadian Guideline Adaptation Study aimed to develop the adaptation process and integrate implementation planning based on actual experience with guideline adaptation.

Descriptive, exploratory case study methods based on Patton’s utilization-focused evaluation framework [13] were used, where evaluation is described as a broad-based gathering of data and information about characteristics, activities, and outcomes (intended and unintended) of a program. The data are then used to make judgments about a program, improve program effectiveness, and/or inform decisions about future programming. Our ‘program’ was the Partnership’s adaptation initiative (*i*.*e*., guideline adaptation approach, resources and facilitation). Each selected group was considered an ‘organizational case’ that served as the unit of analysis. Study cases progressed at their own pace and developed a context-specific approach, *i*.*e*., worked within the available resources/constraints, scopes of practice etc., of their locale.

Specifically, the pan-Canadian study was designed to respond to the following objectives:

1. Map the process and steps undertaken by each Canadian case using mixed methods to describe their experience in guideline adaptation and implementation planning;

2. Discover the range and variation in (internal/external) facilitation, resources and support required to complete the adaptation process;

3. Describe participant and key stakeholders’ experience of the adaptation process, the ADAPTE method, and what other resources and support were required;

4. Ascertain the amount and type of implementation activity/strategies that occurred during the adaptation process.

Ethics approval for this study was obtained from the Queen's University and Affiliated Teaching Hospitals Health Sciences Human Research Ethics Board.

### Procedures

The pilot study case plus an additional four cases self-identified their interest in participating and registered in the Canadian Guideline Adaptation Study. Inclusion was purposeful to represent a broad variation in group characteristics, including jurisdiction, scale of implementation (*e*.*g*., regional, provincial, national), topic, focus of care (*e*.*g*., screening, treatment, supportive care), population (*e*.*g*., pediatric or adult focused), geographic dispersion, and health disciplines (those needed to develop guideline as well as targeted users). Once specific attributes were accommodated and a manageable maximum achieved (n = 5), study registration was closed.

Study cases received support from the Partnership to hire or second an ‘internal’ facilitator/coordinator for their guideline adaptation. The Queen’s team was available to provide training, ‘external’ facilitation and consultation namely, access to knowledge translation and guideline development experts, library science professionals, a study coordinator and data manager (Table 2). Additional tools and data management supports were identified and provided as groups gained experience with the methodology.

**Table 2:** Procedures: Canadian guideline adaptation study

1	Cases received complete ADAPTE Manual and Toolkit (http://www.adapte.org) at outset plus additional and customized tools and resources as needs were determined (based on pilot case experience and on-going support of study cases).
2	Cases completed and submitted on-line ADAPTE Surveys.
3	Case steering committees had access to an orientation session re: ADAPTE methodology plus assistance with ADAPTE Phase 1 Planning via 1/2 or full day workshop facilitated by the Queen’s team.
4	Cases had access to Partnership^1^ supported expertise and resources, e.g., library scientists, methodologists, use of a teleconference line, travel/meeting funds, administrative support, and funds for a part-time ‘internal’ facilitator/coordinator.
5	Cases had access to consultation via teleconference and/or participation in meetings/workshops as needed; average 2-3 sessions per case.
6	Cases agreed to submit project documentation and engage in routine progress checks and calls with Queen’s team.
7	Site visit by Queen’s team project officer at end of ADAPTE Phase 2 included a process evaluation via structured interview and step/tool use audit with study case facilitator/ coordinator and/or chair.
8	Case facilitator/coordinators could participate in an emerging community of practice, the ‘Facilitators’ Network’, via monthly teleconferences supported by the Partnership.
9	Case chairs and facilitator/coordinators participated in a final, full day, face-to-face forum.

^1^ Canadian Partnership Against Cancer.

### Data collection

Study cases provided their meeting notes and project documents, including internal correspondence, and communicated their progress and challenges to the Queen’s team on a regular basis (weekly/monthly) resulting in hundreds of pages per case. Records were kept of all support calls and correspondence with principal case contacts (chair and the internal case facilitator/coordinators). Other data sources included notes and minutes from case orientation and debriefing meetings held with case panels, chairs and facilitator/coordinators, monthly teleconference calls with case facilitator/coordinators, and a full-day, final evaluation forum with case chairs and facilitator/coordinators. Data was often ‘double-captured’ by both the Queen’s team and case participants; data from these multiple sources were summarized in synoptic graphs and tables, including:

1. Case Logs (consolidated meeting notes, case calls, project documents);

2. Case Trajectories (maps of ADAPTE steps taken, sequence and timeline for each case);

3. Case Liaison Records (summary of frequency and type of interaction between each case and Queen’s team);

4. Methodology/Process Audit (detailed step/tool analysis based on a site visit).

Site visits were conducted as the study neared completion. A semi-structured interview with the case chair and facilitator/coordinator included open-ended questions and a checklist to record case perspectives and experience. All chairs and facilitator/coordinators attended a final face-to-face one-day forum, allowing us to share and once more validate our observations and consolidate participant perspectives. Multiple note-takers compared records after the event to identify critical observations. The meeting was audio-taped for field-note verification.

Excerpts from the data management protocol have been included in Additional file 1: Procedures.pdf.

### Data analysis

The extensive documentation and frequent regular contact with case participants afforded a comprehensive overview of case activities. The compiled data were examined to determine stakeholders’ experiences in adapting a guideline, their processes, and the amount and type of implementation activity/strategies undertaken (objectives 3, 4). We conducted text analysis and cross-referenced our multiple data sources to construct detailed case ‘trajectories.’ Each case’s steps and tools used in the adaptation process were audited, followed by a comparative analysis of each to describe their adaptation journey within and beyond ADAPTE. Case logs and trajectories were updated monthly and double-checked by study team members. By continually examining and comparing the experience and path taken by each case, we identified common opportunities to clarify methodology and further develop the process and resources.

## Results

The five cases represented a broad range of guideline interests (Table 3) crossing the developmental spectrum from pediatric to adult focus. Topics were mainly supportive and psycho-social in nature. Some cases’ guidelines applied regionally, whereas others had a national focus. For many participants, motivation to join the guideline group and the Canadian Guideline Adaptation Study was expressed as a means to improve skills and obtain tools to manage research evidence.

**Table 3:** Study case attributes

	**Case 1 (Pilot)**	**Case 2**	**Case 3**	**Case 4**	**Case 5**
**Guideline Title**	Distress Management	Distress Management	Platelet Transfusion	Symptom Triage and Management	Wound Management
**Focus of Health Topic**	*Supportive Care*	*Supportive Care Psychosocial Support (Assessment)*	*Medical Treatment*	*Supportive Care Symptom Management (Remote Support)*	*Supportive Care*
	Diagnosis, referral, and management of distress in adult cancer patients	Management of distress in adult oncology patient with specific focus on assessment	Establishment of platelet transfusion thresholds for pediatric population	Remote support for symptom assessment, triage and management for adult patients undergoing cancer radio & chemo therapy treatments	Skin Care/Wound Management for patients receiving radiotherapy for breast cancer
**Population**	Adult	Adult	Pediatric	Adult	Adult
**Health Disciplines involved**	Multi-disciplinary; primarily front-line caregivers	Multi-disciplinary; primarily specialist services	Oncologists Hematologists	Oncology nurses managing patient symptoms in a home healthcare setting or other environments	Frontline care-givers including oncologists, radiotherapy technicians and nurses
*Developers and Target Users*					
**Jurisdiction**	Provincial	National (pan-Canadian)	National (pan-Canadian)	National (pan-Canadian)	Regional Provincial
*Scale of Implementation*					
**Geographic Area**	Nova Scotia	Canada	Canada	Canada	Manitoba

The cases were also diverse in terms of infrastructure, expertise, funding and access to resources. Typically, cases organized a steering committee and task-oriented ‘working panels’ although members often overlapped and, over the course of the study period (24 months +/−), changes in membership occurred. Pan-Canadian initiatives tended to comprise larger groups (representing multiple regions, provinces, agencies) and included ‘co-chairs’; size ranged from 8 to 15 participants. Working panels included members with some experience in previous guideline development initiatives (*e*.*g*., content experts, AGREE [14] users), and novice members who had little guideline development experience. All included multiple disciplines ranging from front-line caregivers to specialist services. Some panels involved a patient member during planning phases, but most indicated they would seek patient input at the guideline review/endorsement stage. Unlike most dedicated guideline enterprises, but similar to most end-users of guidelines, our groups were 100% volunteers working over-and-above full-time clinical and/or administrative responsibilities and frequently facing significant time constraints, variable skills sets, and limited administrative support.

Panels with a national focus faced unique challenges given the provincial/territorial organization of healthcare in Canada. Variation in regional practices was common, and they had to organize and operate where no national body had previously existed. Their first task was to establish themselves as a national guideline entity and negotiate fundamental issues of authority, mandate and infrastructure, an often time-consuming and arduous task. For example, a group working on ‘distress assessment’ had interests from multiple disciplines (social work, nursing, psychology); their respective professional associations had shared responsibility for standards and practice, yet none was mandated to develop guidelines.

### Process and steps undertaken in guideline adaptation and implementation planning

**Figure 2:**
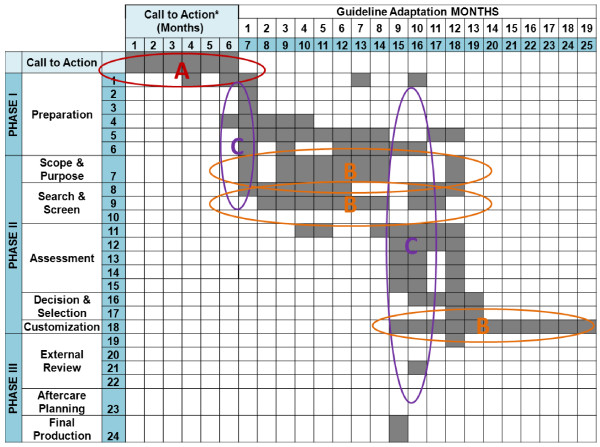
Representative case trajectory: emerging patterns using the ADAPTE methodology.

At 24 months, the majority (three of five cases) had largely completed their guidelines and were at the external review phase. Several consistent features emerged and novel aspects were discovered when we examined the ADAPTE steps used by each case. A typical case trajectory (Figure 2) plots three defining elements observed across cases: a call-to-action phase, the non-linear and iterative nature of steps, and the synchronization of some activities.

### Call-to-action

Although the ADAPTE methodology begins with a ‘Set-Up’ phase and preparation module, the scope of activity appears premised upon some level of guideline development infrastructure and an agreed upon, reasonably well-focused initiative. By comparison, most of our cases lacked this foundation, and without a dedicated or formal guideline panel, required more time, effort and support to begin. This ‘call-to-action’ phase is a new element in the Canadian experience.

Field-testing revealed that this step required complex planning and needed to be positioned early in the process (pattern A). As many as four to six months were required for new groups to articulate objectives, agree on mandate, identify and engage key stakeholders, and secure resources. Challenges included: establishing clinical or jurisdictional authority where none existed previously and where institutional, provincial and national interests needed clarification and alignment; confusing or conflicting information regarding clinical priorities or a lack of baseline data; absence of established communication channels; proceeding with guideline directives in the face of significant resource constraints (*e*.*g*., economic, expertise). These issues were particularly concerning to pan-Canadian guideline initiatives. Given the many requests for assistance with start-up, we restructured and expanded this element, naming it the ‘Call-to-Action’ step, to which orientation, training and facilitation supports were added to help groups better navigate ‘pre-adaptation’ essentials.

### Iterative activities

Case participants reported that the work sequence set out in ADAPTE was neither straightforward nor linear. Less experienced groups in particular moved back and forth in the ‘Search and Screen’ step between defining their health question(s), searching for evidence, and further refining the questions based on search findings. Drafting, reviewing and revising the adapted guideline reflected another lengthy cyclic period of activity (pattern B).

### Synchronization of activities

Cases commonly engaged in several stages and steps simultaneously during the adaptation (pattern C). Those managing more complex guidelines often had unfinished business in one phase while they progressed onto tasks in the next phase; *e*.*g*., they expanded a search for selected evidence while reaching consensus on other evidence, or began drafting recommendations and application tools for one aspect of the guideline while continuing to review evidence for other sections. A key factor influencing the cycling and timing of activities was time to completion (up to two or more years). Long timelines often resulted in changes to panel composition or institutional leadership that created delays, repetition of effort, or changes in plan.

### Facilitation activity, resources and support

Facilitation was provided both internally (local staff, case-based) and externally (Queen’s team) and ranged from supplying task-focused assistance, to developing group skills, to methodological aspects.

Internal facilitator/coordinators possessed diverse backgrounds in quality assurance, project management, administration, education, information technology, and clinical practice. They had a ‘working knowledge’ of levels of evidence and appraisal requirements. These part-time (0.25 to 0.5 FTE^a^) internal facilitators fulfilled a coordinating role during adaptation and acted as the primary interface with the Queen’s team. Internal facilitators reported that the administrative aspects of the position were especially demanding [12]. Specifically, they contributed to management of their respective working panel and agency communications, organized meetings, liaised with information science professionals and methodologists, retrieved guidelines and documented search and screen decisions, managed AGREE appraisals, prepared recommendations matrices, facilitated consensus, prepared guideline drafts, and coordinated internal and external reviews of the guideline. Internal facilitators identified the importance of a ‘project management’ approach in facilitation.

The locally designated facilitators developed close working relationships with their initiative chairs. Facilitation activity often went beyond what they individually contributed because they shared work with steering committee and working panel members. As such, facilitation was perceived as a process engaged in by a number of individuals rather than a specific single role. However, facilitators uniformly noted the need for an explicitly identified, responsible individual: as articulated by one facilitator, ‘someone does have to coordinate the whole thing.’

Internal facilitators also considered it important for their steering committee and working panel members to ‘have access to a venting office’ for problem-solving and advice, as well as to maintain momentum of the work (referred to as the ‘cheerleader aspect’). It was perceived as a sometimes lonely job requiring knowledge and a wide-ranging set of skills and attributes, such as effective communication, organization, group dynamics and leadership ability, which they referred to collectively as ‘relational practice skills.’

As external facilitators, the Queen’s team provided each case with an orientation workshop addressing fundamentals of evidence-based practice, the Knowledge-to-Action cycle, and the phases, modules, steps and tools of the ADAPTE method. During these orientation workshops, steering committee and working panel members (often coming together for the first time) developed a shared understanding of objectives and process. Lively debates revealed differences in perceptions, occasionally on fundamental concepts: ‘I think of it as a wish list of how we would provide care if we had endless resources at our disposal… It’s just a guideline; we don’t have to follow it… What is the difference between a guideline and a policy? … Will we be liable for following these recommendations?… How much flexibility do we have?’

All cases expressed concern about undertaking the process independently for the first time. Cases were assisted by the Queen’s team during the ‘Set-Up’ phase of ADAPTE with skills and resource assessments, and development of clinical questions. Regular coaching and methodological consultation was provided in response to specific case requests or when they appeared overwhelmed with the process. We were frequently contacted to clarify ‘how-to’ aspects related to individual steps and tasks. While the ADAPTE process, manual and toolkit provided a useful starting point, we responded to case-identified needs to adapt the ADAPTE methodology by adding Canadian content or providing new tools (Table 4). A paradox existed in which the ADAPTE method and materials were perceived as being both ‘too comprehensive’ but ‘not detailed enough.’

**Table 4:** Evolution of ADAPTE method and materials

**1**	**Modifications and additions to process and supports, e.g.,**
	Heightened focus with more information/guidance on implementation and facilitation aspects
	Modification of task and sequence, e.g., addition of the pre-adaptation Call-to-Action step, placement of PIPOH^1^ forward in the process, an explicit implementation planning component, reduction in total number of steps
	Modification of existing tools to reflect Canadian content, e.g., Canadian guideline sources
	Addition of orientation, training, and methodological support, e.g.,
	- orientation workshop agenda with support materials including PowerPoint presentation slide decks, discussion guides (e.g., ‘What is a Guideline?’) and planning activities
	- new *Library Science Supplement*: a detailed, basic guide to designing and executing a search strategy, prepared by Queen’s team Library Scientists
	- expanded index and links to guideline development and evidence-informed practice resources
	- expanded information on tasks and techniques, e.g., consensus processes, evidence grading methods
**2**	**Multiple, new project management and documentation tools and supports, e.g.,**
	Skills and resources needs assessment checklist
	Terms of reference templates for steering committees and working panels
	Spreadsheets to manage search citations and screening decisions
	Facilitator’s guide to managing AGREE appraisals
	Template letters to invite/instruct AGREE raters, contact source developers
	Spreadsheets to manage consolidation of AGREE and other appraisal data
**3**	**Enhanced navigation, access, and ease of use, e.g.,**
	Provision of interactive, electronic tools and templates (e.g., reformatted PIPOH, Recommendations Matrix, data summary tables); revised indexing and links to tools and resources
	Addition of a *Quick Reference Guide*, *Progress Checks*, and decision cues
	Multiple *Field Notes* to describe real challenges/solutions experienced by the Canadian Guideline Adaptation Study cases
NOTE: the final version of **CAN-IMPLEMENT** substantively extends the methodology and user supports. Guideline adaptation is embedded in the Knowledge to Action cycle as part of a new three phase model:	
- PHASE 1 Identification and Clarification of Practice Issue/Problem	
- PHASE 2 Solution Building	
- PHASE 3 Implementation, Evaluation and Sustainability	

^1^ PIPOH = P- population, I- intervention(s), P- professionals, O- outcome(s), H- healthcare setting.

On topics where few guidelines were available, we assisted cases to search for systematic reviews and offered tools/ supports for appraising systematic reviews, *e*.*g*., Assessment of Multiple Systematic Reviews (AMSTAR) [15]. Most cases struggled with differences in evidence classification systems. We provided information about leading approaches and assisted in translating levels of evidence across systems. Cases also sought guidance with formal and informal consensus methods (*e*.*g*., Delphi techniques [16-18]). They found the ADAPTE ‘Acceptability and Applicability’ worksheet limited in scope and were encouraged to explore additional tools, *e*.*g*., the Guideline Implementability Appraisal (GLIA) instrument [19].

Convenient electronic access to materials and supports was vital for the Canadian context given the broad geographic dispersion even in one region of the country. It was necessary to reformat ADAPTE and other tools for electronic sharing. In addition, the Queen’s team assisted some cases with managing their data entry and project documents online. This was especially important for pan-Canadian initiatives that relied on email or Internet workspaces and teleconferences to keep communications and travel costs to a minimum.

Queen’s team members were often invited to case teleconference calls or contacted directly to advise on process, strategies, challenges, or simply to act as a sounding board. This was seen as particularly valuable by the case facilitator/coordinators and prompted the development of a national Guideline Facilitators’ Network that featured a monthly teleconference enabling participants to share guideline activities, challenges and successes. This forum has continued beyond the project.

Facilitation was clearly a ‘key ingredient’ and the scope of enquiry in year two was expanded to focus specifically on the development of a conceptual framework and taxonomy for facilitation. An integrative review, case audit and structured interviews, and an international two-day facilitator’s forum were used to delineate key practical and conceptual elements of facilitation as a ‘process beyond an assigned role’ in implementation [11,12,20]. Facilitation is described as a multifaceted process comprising more than 50 specified actions grouped across four stages: planning for change, leading and managing change, monitoring progress and on-going implementation, and evaluating change.

### Participant experience and perspectives

Overall, cases perceived the process of adaptation as enormously time-consuming. There was a palpable tension between the need for efficiency and the demand for rigour. Although cases initially wanted to ‘streamline’ and ‘simplify’ the 3-phase, 9-module, 24-step ADAPTE methodology, they were equally concerned that the integrity of the methodology be maintained. Practitioners identified they were not necessarily expert developers and that their ‘steep learning curve’ contributed to the lengthy time needed to complete activities. Nonetheless, they explored and tried strategies to reduce or consolidate steps and refine the process to create evidence-informed recommendations in a timelier manner (Table 5).

**Table 5:** Study participant ideas for streamlining process

1	Limit guideline scope; reduce number of clinical questions.
2	Reduce duplication by forming collaborative groups.
3	Engage specialists and methodological expertise when needed (e.g., library science, evidence appraisal).
4	Find efficiencies in searching and screening the literature, e.g., limit inclusion to previously reviewed and quality-appraised guidelines.
5	Consult source developers earlier in the process to verify evidence and pending updates.
6	Limit the size, representation and involvement of steering committees and working panels and convene only when strategic decisions or consensus are needed.
7	Prioritize and delegate some of the methodology - not all panel members need to be engaged in every step and activity.
8	Simplify the presentation of evidence and assessments for discussion and consensus management, e.g., distribute summaries vs. raw appraisal data/scores to decision-makers.

During a final evaluation forum, case chairs and facilitators reported a new appreciation for the importance of transparency and meticulous documentation and acknowledged that this had been a weakness in their previous guideline development efforts. This pertained particularly to their record of the literature search, screening protocol and selection rationale, preparation of the ‘Recommendations Matrix’ including source and grade of supporting evidence, and reporting of their consensus processes and decisions.

Case participants commented on the positive impact of participating in the adaptation process, stating it had sparked a ‘groundswell of talk, activity and thinking about evidence-based practice’ within their organizations. They attributed important educational and professional development outcomes to their inclusion in the study.

These naturally formed groups became part of an active partnership between healthcare practitioners and researchers, a collaboration they perceived to be instrumental to guideline adaptation, and more importantly, implementation planning. This inclusive, participatory approach was viewed as an important ‘community of practice’ which advanced the adaptation process by providing access to a broad range of skills and experience, ensuring practice issues were understood and addressed as they surfaced, and giving groups who were not formal guideline developers, a deeper appreciation of the need for a rigorous, transparent process. They also indicated that the collaboration was a powerful ‘external lever’ that added credibility to their guideline initiatives within their organizations.

Because available guidelines did not respond to all of their questions, participants discovered that guideline adaptation as a strategy has its limits. Four of the five cases undertook hybrid guideline projects involving both adaptation and de novo elements. When source guidelines were unavailable, out-of-date, or did not adequately align with the specified health questions, searching and updating primary study evidence was required. More extensive search, appraisal and synthesis demanded more time and methodological skill than they had envisaged or were equipped to manage.

An unexpected finding was how enthusiastically cases embraced the conceptual underpinning for knowledge translation. We introduced the KTA framework at orientation sessions to highlight how guidelines contribute to evidence-informed practice, but did not foresee cases actively using it to ‘situate their work’ of adaptation in their own discussions with practitioners, administrators and decision-makers. Case chairs and facilitator/coordinators reported using it effectively to guide discussions about implementation issues such as barriers, facilitating factors, and ideas on tailoring implementation strategies.

### Implementation activity and strategies during guideline adaptation

An important observation was how, in practical terms, planning for implementation began both implicitly and explicitly from the outset with guideline adaptation. Cases undertook the process by viewing the practice issue from the perspective of potential solutions for their own local context. They planned and conducted preparatory activities such as needs assessments and environmental scans. Innovative strategies designed by the cases to plan for implementation included polling constituents regarding current policies, practices, and priorities, proactively communicating guideline planning activities and progress, and including allied health services on guideline working panels. They were strategic in selecting steering committee and working panel members and in their communications, *e*.*g*.: including information technology staff early in the process to facilitate anticipated modifications to the health record; ensuring that steering committee members represented national interests and were included as authors on publications derived from the initiative to secure future endorsement and ownership of the recommendations.

Constructive discourse on applicability and acceptability of guideline recommendations was continuous throughout the process; *i*.*e*., this activity was not contained within a single step, task or tool. Panel members, especially front-line care providers, were often able to anticipate facilitating factors or raise concerns about potential barriers to the uptake of the proposed recommendations.

## Discussion

The Canadian Guideline Adaptation Study examined how groups used a ‘planned action’ approach to integrate knowledge created outside their context with local practice and system requirements. This multiple-case series provides the first comprehensive field study of guideline adaptation and implementation planning. In an approach that was formative rather than prescriptive, we provided basic support and facilitation in adapting a guideline and planning for implementation, to fundamentally understand how groups naturally undertook the process.

Five cases embarked on guideline adaptation and implementation planning related to an identified issue in their context. We observed an active focus on implementation planning during the adaptation process, particularly in regards to assessment of barriers and facilitating factors. The process of adaptation involved considerably more than simply having an available methodology. Adaptation took longer than expected (12 to >24 months) and was more complex than many had anticipated and required substantive support and facilitation.

The findings of this Canadian initiative led to the creation of a structured process and user guide called the ‘CAN-IMPLEMENT Guideline Adaptation and Implementation Planning Resource’ [21]. The ADAPTE methodology provides valuable support for adaptation especially for well-resourced guideline panels but, in our experience, could not be used as a stand-alone resource for less experienced groups. Our analysis of case experience with guideline adaptation led to reorganization and expansion of the initial ADAPTE methodology to a new three-phase approach addressing a full knowledge application cycle. The CAN-IMPLEMENT resource purposely addresses practical, ‘how-to’ aspects to assist those engaged in adapting existing guidelines for implementation. It uniquely targets novice developers by expanding tactical support to the leaders and managers of guideline adaptation panels framing the adaptation process within a broader conceptual framework for knowledge translation. Although adjustments were made to tasks and sequence, ADAPTE’s foundational rigor and elements remain intact.

### Limitations

Data for the study came from multiple documentary and interview sources, including self-reports. Given our role as supportive observers, some risk of ‘social desirability response’ existed, *i*.*e*., the provision of information participants think investigators want to hear about the process under evaluation. However, given the frank and often negative issues brought forth and our complementary and overlapping data sources, we believe this was not an issue. Interpretation of the subjective data might be seen as a limitation but having data provided by both participants and the facilitators (internal and external) increases our confidence in its fidelity.

The results of this study relate to five Canadian groups working in cancer care who undertook guideline adaptation. Their experience could be different from those in other countries or in other areas of healthcare. Although results may not be generalized, the variety of groups, topics, stages of readiness, and experience improves the likelihood that findings are transferrable.

It should be recognized that the ‘pan-Canadian’ adapted guidelines generated would likely need further adaptation at a local (provincial or institutional) level; it was not possible within the parameters or timeframe of this study to follow this activity.

## Conclusions

This study provides valuable information for those interested in adapting existing guidelines. We found that there is a steep learning curve and a substantive requirement for methodological and facilitation support, especially for newly formed or less experienced groups undertaking guideline adaptation and implementation planning. There was, however, major benefit to active participation of front-line care providers in the contextualization and ownership of the recommendations. In this study, front-line practitioners gained confidence in describing and defending changes for best practices as a result of participating in the process.

Managing and facilitating guideline adaptation and implementation is a demanding and time-consuming process. Panels are cautioned to realistically determine workload, resources, access to methodological expertise, and the need for a dedicated project coordinator/facilitator. Multiple social, economic and political variables will influence the effectiveness of guideline ‘uptake’ and outcomes, and groups are encouraged to ensure institutional commitments and resources are in place. In summary, our recommendations are:

1. A guideline adaptation process should address two elements: the process (engagement and capacity building) and a quality product (an adapted guideline).

2. Guideline adaptation should not be considered as an episodic activity but part of a continuum in the evidence-informed practice journey and be planned for accordingly.

3. Orienting the guideline adaptation process within a planned-action framework is useful as a roadmap to embed guideline adaptation in the larger activity of evidence use and to assist guideline adapters/users to plan effective adaptation, implementation and evaluation of outcomes.

CAN-IMPLEMENT is available at http://www.cancerview.ca[21]. We welcome reader feedback on the CAN-IMPLEMENT process and resources.

## Endnote

^a^FTE = Full Time Equivalent

## Competing interests

The authors declare that they have no competing interests. IDH and MBH developed the practice guideline evaluation and adaptation cycle (PGEAC) [22] and were founding members of the ADAPTE Collaboration.

## Authors’ contributions

MBH was principal investigator on the study and responsible for conceptualization, ethical approval, the conduct and management of the study, and analysis and interpretation of the data. IDG was a co-investigator; JvdH was senior project officer, and both contributed to the study conceptualization, analysis and interpretation of data. EJD was responsible for the analysis of the facilitation elements. MEC was responsible for data management and contributed to the analysis. VA was responsible for study coordination and contributed to the evaluation design and analysis. All authors have read, edited and approved the final manuscript.

## Supplementary Material

Additional file 1 Procedures. Click here for file
