# Supplementary material for: Guideline adaptation and implementation planning: a prospective observational study
Source: Implement Sci. 2013 May 8;8:49. doi: 10.1186/1748-5908-8-49 (PMC3668213; doi:10.1186/1748-5908-8-49)
Supplement: Additional file 1 — Procedures. [file 1748-5908-8-49-S1.pdf]

(Excerpts)

August 15, 2008

Guideline Adaptation Procedures Manual

Data Management Protocol and Study Instrument Variable Information

Canadian Guideline Adaptation Study

An evaluation of guideline adaptation and implementation
using the structured methodology, ADAPTE

('CAN-ADAPTE' = project working title)

Part II: Overview of Case Data Management Protocol

Data collection

The Project Coordinator and Project Assistant collect case data using the following methods: email correspondence, meeting and field notes, and interviews (formal and informal). Each case has been allocated a binder in which data will be stored. The Project Assistant is responsible for maintaining case binders and ensuring that they are up to date. All source documents are contained in the case binders. The Project Coordinator and Project Assistant meet monthly to review the activity of each case and ensure that the Project Assistant has collected all new case data.

Case tracking documents

Four documents have been developed in order to track individual case activity: Case Log (pg. 63), CAN-ADAPTE Trajectory (pg. 64), Case Liaison Record (pg. 65), and CAN-ADAPTE Methodology Process Audit (pg. 66).

1. **Case Log**
   Objective: To summarize in detail case activity, including date of activity, estimated duration, number of participants, name and title of participants
   Data Sources: Meeting and teleconference notes, minutes, field notes, email communications, project documents (i.e. PIPOH, work plan, etc.)
   Accountability: Project Assistant
   Maintenance: Update monthly

2. **CAN-ADAPTE Trajectory**
   Objective: To plot case progress on a monthly timeline using CAN-ADAPTE guideline adaptation methodology (per Phase, Module and Step)
   Data Sources: Case Log
   Accountability: Project Assistant & Project Coordinator: The Project Assistant and Project Coordinator review the Case Log to reach consensus on which phases, modules and steps, the case has completed and is working on, as well as the duration and sequence of each phase, module and step; The Project Assistant is responsible for updating the CAN-ADAPTE Trajectory
   Maintenance: Update quarterly

3. **Case Liaison Record**
   Objective: To record chronologically the Guideline Adaptation team relationship and interaction with each case, and identify level of consultation, facilitation, methodological and project management support
   Data Sources: Case Log
   Accountability: Project Assistant
   Maintenance: Update quarterly

4. **CAN-ADAPTE Methodology Process Audit**
   Objective: To record in detail case engagement with each Step, Tool (Resource) and the Sequence used, with supporting details and comments/case feedback
   Data Sources: Case Log, Surveys, structured interviews
   Accountability: Project Coordinator: Using the Structured Interview Guide: Case Tracking: Steps, Tools, Sequence to conduct the interview (pg. 69), the Project Coordinator conducts an interview with key informants at end of each Phase in methodology. The interview is scheduled at the key informant's convenience.
   Maintenance: Update per phase

Appendix II: Case Tracking Documents

Case Log

Case Title

Date	Activity	Estimated Time (in hours)	Number of Participants	Name	Title	Summary	Facilitation

CAN-ADAPTE Trajectory

Month 1 - Oct'07	Month 2 - Nov'07	Month 3 - Dec'07	Month 4 - Jan'08	Month 5 - Feb'08	Month 6 - Mar'08	Month 7 - Apr'08	Month 8 - May'08	Month 9 - Jun'08	Month 10 - Jul'08

Call to Action
1) …
2) …
3) …

CAN-ADAPTE Phase I, Preparation Module
M4-7: Steps 1-6

CAN-ADAPTE Phase II, Scope & Purpose Module, Step 7

CAN-ADAPTE Phase II, Search & Screen Module, Step 8

Case Liaison Record

**Activity/ Title**	**Oct-07**	**Nov-07**	**Dec-07**	**Jan-08**	**Feb-08**	**Mar-08**	**Apr-08**	**May-08**	**Jun-08**	**Jul-08**	**Aug-08**	**Sep-08**
Raised & accepted as potential project	X Oct 2											
Correspondence to set-up teleconference call with Project Leads	X Oct 18											
Teleconference with Project Leads re: collaboration		X Nov 2										
Sent ADAPTE manual & toolkit to Project Leads		X Nov 2										
Provided consultation to Project Leads			X Dec 20									
Correspondence to set-up next teleconference				X Jan 5								
Face to face meeting with Steering Committee					X Feb 10							
Sent Local Facilitator CAN-ADAPTE materials					X Feb 11							

CAN-ADAPTE Methodology Process Audit
PROJECT:

	Module	Step	Y/N	Tool	Ref. Page	Used Y/N	Date Started	Date Completed	Process Details
**Phase I – Planning & Set-Up**	***Preparation***	1. Establish an Organizing Committee, working panel, resource team etc …		Guideline Development and Implementation Resources<br><br>CAN Training Materials Reference<br><br>CAN 24 Steps Summary<br><br>CAN Tools Reference	Pg. 9<br>Tool 1				
		2. Select a topic using criteria		PIPOH Checklist<br><br>CAN PIPOH Instrument	Pg. 16<br>Tool 6				
		3. Check if adaptation is feasible …		Guideline Search Sources & Strategies<br><br>CAN What is a Guideline? Information Sheet<br><br>CAN Guideline Sources Checklist	Pg 12<br>Tool 2				
		4. Identify necessary resources and skills		CAN Skills Assessment Checklist<br><br>Resources on Consensus Process	Page 13<br>Tool 4				
		5. Complete tasks for set-up							
		6. Write the adaptation plan		Work Plan Example<br><br>CAN Adaptation Work Plan<br><br>Declaration of Conflict of Interest	Pg 15<br>Tool 5<br><br>Pg 13<br>Tool 3				

	Module	Step	Y/N	Tool	Ref. Page	Used Y/N	Date Started	Date Completed	Process Details
**Phase II - Adaptation**	***Scope & Purpose***	7. Determine health q…		PIPOH Checklist<br><br>CAN PIPOH Instrument	Pg. 16<br>Tool 6				
	Search & Screen	8. Search for Guidelines and other relevant documentation		Table for Recording Guideline Characteristics<br><br>CAN Tips & Tools: Search Strategies & Mgt of Citations	Pg. 19<br>Tool 7				
		9. Screen retrieved guidelines..		Table for Recording Guideline Content	Pg. 19<br>Tool 8				
		10. Reduce guidelines (using rigour dimension of AGREE)		AGREE Instrument (limited assessment, e.g. rigour only)<br><br>AGREE Inter-rater & Calculation Spreadsheets<br><br>CAN AGREE Package	Pg. 20<br>Tool 9<br><br>Pg. 20<br>Tool 10				
	Assessment	11. Assess guideline quality		AGREE Instrument (full assessment – all domains)	Pg. 22<br>Tool 9				
		12. Assess guideline currency		Sample Currency Survey	Pg. 25<br>Tool 11				
		13. Assess guideline content		Sample Recommendations Matrices	Pg. 27<br>Tool 12				
		14. Assess guideline consistency		… Assessing the Quality of Study Search & Selection<br><br>…Recording Evaluation of Consistency…	Pg. 28<br>Tool 13<br><br>Pg. 29<br>Tool 14				
		15. Assess acceptability & applicability of recommend'ns		Worksheet: Acceptability/ Applicability	Pg. 29<br>Tool 15				
	Decision & Selection	16. Review Assessment results from steps 11-15							
		17. Achieve consensus to Reject, Accept or Modify Guideline(s)		no tools for achieving consensus, sample process described pg. 33					

	Module	Step	Y/N	Tool	Ref. Page	Used Y/N	Date Started	Date Completed	Process Details
	Customization	18. Prepare draft adapted guideline		Checklist of Adapted Guideline Content<br><br>Report on Results of Updating Process	Pg. 34 Tool 16<br><br>Pg. 34 Tool 17				
**Phase III - Finalization**	*External Review and*	19. External Review…		18. External Review Survey	Pg. 36 Tool 18				
		20. Consult with endorsement bodies							
		21. Consult with source guideline developers							
		22. Acknowledge source documents							
	Aftercare Planning	23. Plan for aftercare of the adapted guideline							
	Final Production	24. Produce final guideline document							

Structured Interview Guide - Case Tracking: Steps, Tools, Sequence

Introduction and preparation:

1. Indicate that purpose of this interview/discussion is to determine - at detailed, step-by-step level - the activities and individual tools/resources actually undertaken and used by each group in following the (CAN) ADAPTE guideline adaptation methodology.

2. Indicate you have tentatively completed information based on previous communications and understanding of project to date (note: provide partially completed record in advance of interview, preferably 2-3 days). Advise that you would like to confirm details, fill in any gaps, and note any issues or challenges at each phase, module or step of process. You also want to determine how often or how many times it is necessary to return to any particular step, acknowledging that, for most Study cases, process has not been linear.

3. Advise that it will help to have their copy of ADAPTE Resource Manual and Tool kit, including CAN-ADAPTE Supplementary Resources available for reference as well as any meeting or planning notes, emails and documentation related to the project.

4. Advise it should take 30 minutes (per Phase); we can schedule an interview per phase or complete as much as possible depending on case progress and informant's time.

5. Explain you would like to record call to assist with note taking and keep informant's time to a minimum. Gain permission to record.

6. Describe "audit" tool - grid/tracking record - content and layout.

7. At close of interview, thank informant and offer to provide copy of completed record.

Note: for each Phase/Module/Step …

1. Refer to ADAPTE Manual; remind informant of the tasks involved at each Phase, Module and Step

2. Use CAN-ADAPTE 24 Steps Reference and 18 Tools Reference summaries to guide discussion.

Questions:

1. Did your group engage in this particular activity/Phase/Module/Step of the process?

2. When do you think this Phase/Module/Step started? Has it been completed? When was it completed?

3. Did your group follow the advice/recommendations provided in the Manual and Resource Kits? Who had access to the materials? How was information communicated to the group?

4. Were you able to effectively complete each and every task within this Phase/Module/Step? Were tasks or items excluded? Which activities were most useful?

5. Do you recall using any of the supporting tools for this Phase/Module/Step from the ADAPTE and CAN-ADAPTE kits; how were they used; who used them?

6. Were the materials provided helpful to the group in completing this Phase/Module/Step?

7. What else would you have found helpful at this point?

8. What were some of the key challenges in completing this Phase/Module/Step? And key successes?

9. Have you had to return to this Phase/Module/Step? Describe how and when.

10. What would you do differently if you were starting at this point again?

Queen's University

CANADIAN PARTNERSHIP AGAINST CANCER | PARTENARIAT CANADIEN CONTRE LE CANCER

AGENDA: Evaluation Study Final Forum

0830 Breakfast

0900 Welcome and Introductions; outline of the day

0915 CAN-ADAPTE Process and Evaluation Study Findings

10:15 Break

10:30 CAN-ADAPTE: Facilitation Study report

11:00 De-briefing with teams: using SLOT Analysis

12:00 Final ADAPTE Survey form – Case Teams

12:30 Lunch

1:30 Presentation and Discussion: Communities of Practice - Network Analysis report

2:30 Preview of Next Steps:

i. new model for adaptation
ii. modified CAN-IMPLEMENT Resource
iii. next steps – Implementation resources

3:00 Wrap-up and Adjourn

Discussion Guide: Evaluation of Guideline Adaptation program

Using a Strengths, Limits, Opportunities, Threats (SLOT) framework, consider:

1. Overall perspective regarding participation in CPACC guideline adaptation process?

2. What worked well; what supports were helpful?

3. Barriers or limitations encountered; suggestions for improvement?

4. How will you use this experience in future initiatives?

5. Specific to the(ADAPTE) adaptation process:

    - Value of process
    - Clarity, comprehensiveness, usefulness of resources
    - Comments on individual phases, modules, steps or tools

6. (Final ADAPTE Survey : one form per Study case)
